# Efficacy and safety of single- and double-dose intravenous tranexamic acid in hip and knee arthroplasty: a systematic review and meta-analysis

**DOI:** 10.1186/s13018-023-03929-9

**Published:** 2023-08-10

**Authors:** Yong-Ze Yang, Qing-Hao Cheng, An-Ren Zhang, Xin Yang, Zhuang-Zhuang Zhang, Hong-Zhang Guo

**Affiliations:** 1 First Clinical Medical College of Gansu, University of Traditional Chinese Medicine, Lanzhou, China; 2 People’s Hospital of Gansu Province, 204 Donggang West Road, Chengguan District, Lanzhou, 730000 China

**Keywords:** Arthroplasty, replacement, hip, Arthroplasty, replacement, knee, Tranexamic acid, Single dose, Double dose

## Abstract

**Objective:**

With the increasing prevalence of osteoarthritis of the hip and knee, total joint replacement, the end-stage treatment, provides pain relief and restoration of function, but is often associated with massive blood loss. Tranexamic acid (TXA) has been reported to reduce perioperative blood loss in hip or knee arthroplasty. However, the optimal dose of TXA administration remains controversial. Therefore, we performed a meta-analysis combining data from 5 trials comparing the efficacy and safety of one fixed dose of 1 g intravenously administered TXA with two doses of 1 g each administered intravenously for hip or knee arthroplasty.

**Methods:**

PubMed, Medline, Embase, Web of Science, and The Cochrane Library were searched from January 2000 to February 2023. Our meta-analysis included randomized controlled trials and cohort studies comparing the efficacy and safety of different doses of intravenous TXA (IV-TXA) for THA or TKA. The observation endpoints included total blood loss, postoperative hemoglobin drop, blood transfusion rate, length of hospital stay, incidence of deep venous thrombosis (DVT), and incidence of pulmonary embolism (PE). Meta-analysis was performed according to Cochrane's guidelines and PRISMA statement. The Danish RevMan5.3 software was used for data merging.

**Results:**

Five cohort studies involving 5542 patients met the inclusion criteria. Our meta-analysis showed that the two groups were significantly higher in total blood loss (mean difference (MD) = − 65.60, 95% confidence interval (CI) [− 131.46, 0.26], *P* = 0.05); blood transfusion rate (risk difference (RD) = 0.00, 95% CI [− 0.01, 0.02], *P* = 0.55); postoperative hemoglobin (MD = 0.02, 95% CI [− 0.09, 0.13], *P* = 0.31); postoperative hospital stay days (MD = − 0.13), 95% CI [− 0.35, 0.09], *P* = 0.25); DVT (RD = 0.00, 95% CI [− 0.00, 0.01], *P* = 0.67); PE (RD = 0.00, 95% CI [− 0.01, 0.00], *P* = 0.79). There was some inherent heterogeneity due to variance in sample size across each major study.

**Conclusion:**

1 dose of 1 g and 2 doses of 1 g IV-TXA each time have similar effects on reducing blood loss, blood transfusion rate, postoperative hemoglobin level, and postoperative hospital stay after TKA or THA, without increasing the risk of postoperative complications risk. For patients at high risk of thromboembolic events, one dose of 1 g TXA throughout surgery may be preferred. However, higher-quality RCT is needed to explore the optimal protocol dose to recommend the widespread use of TXA in total joint arthroplasty.

*Trial registration* We conducted literature selection, eligibility criteria evaluation, data extraction and analysis on the research program registered in Prospero (CRD42023405387) on March 16, 2023.

**Supplementary Information:**

The online version contains supplementary material available at 10.1186/s13018-023-03929-9.

## Introduction

Due to the aging population, there has been a significant increase in the number of patients undergoing total hip arthroplasty (THA) and total knee arthroplasty (TKA) in recent years [1, 2]. In 2017 alone, 500 million people worldwide were reported to be affected by osteoarthritis, with 61 to 2 million in China alone [3]. The incidence of the disease increases with age, particularly in people over 65 years old, where the prevalence is as high as 50% [4]. The increasing life expectancy of the population is contributing to the rising prevalence of osteoarthritis [5]. Effective management of osteoarthritis has become crucial for improving the quality of life of middle-aged and elderly people. THA and TKA have been used with considerable success for pain relief and functional recovery in end-stage knee and hip disease. However, controlling the incidence of postoperative complications after THA and TKA has become a hot topic of research in recent years. Several studies have shown that the incidence of postoperative complications is associated with massive perioperative blood loss and transfusion [6–8]. Blood transfusions not only increase the risk of immune response and disease transmission but also the risk of postoperative and intraoperative complications and the cost of hospitalization [9]^.^ To address these issues, tranexamic acid (TXA) has been widely used in the perioperative period for THA and TKA due to its ability to control intraoperative bleeding [10].

TXA is a synthetic amino acid derivative that inhibits fibrinolysis by reversibly blocking the lysine binding site on the fibrinogen molecule and inhibiting the conversion of fibrinogen to fibrinolytic enzymes [11, 12]. This inhibits fibrinolysis. Numerous studies have shown that intravenous application of TXA is effective and safe in the treatment of TKA and THA [13–17]. THA and TXA can be administered intravenously, topically, orally, and in combination [18]. The most common of these is by intravenous drip [19]. The biological half-life of TXA in the joint fluid is approximately 3 h [11, 20]. Many studies have reported the use of different doses of TXA in the perioperative period for THA and TKA to clearly achieve better results [21–24]. At the same time, there is a theoretical concern due to its antifibrinolytic activity and the risk of non-negligible thromboembolic complications. There is no unanimous opinion on the efficacy and safety of intravenous tranexamic acid (IV TXA) in relation to its delivery strategy to reduce blood loss after arthroplasty. In the Mohammad meta-analysis, it was reported that high doses of IV TXA (≥ 2 g or ≥ 30 mg/kg as a single push) reduced transfusion requirements compared to standard doses (≤ 1 g TXA), but the effect on thromboembolic events and mortality was uncertain [25]. In a study by A Fígar et al., a single dose (1 g) of intravenous tranexamic acid was found to reduce transfusion after total hip arthroplasty without increasing the incidence of adverse events [26].

There is currently no evidence to support the efficacy and safety of a double-dose (1 g per dose) intravenous TXA regimen compared to a single-dose (1 g per dose) intravenous TKA regimen. Studies conducted on patients who did not receive TXA as a control group have demonstrated the effectiveness of the single-dose regimen in preventing perioperative blood loss [27–29]. However, other studies comparing a single dose with two doses have shown some efficacy in preventing perioperative blood loss. Nevertheless, studies comparing a single dose with two or more doses have yielded different results [30–32]. In the Sershon study [33], all TXA groups tested, including the single-dose (1 g) IV TXA group and the double-dose (2 g) IV TXA group in the randomized controlled study, exhibited similar blood-sparing characteristics in total hip arthroplasty revision. The results suggest that no significant differences were found between the TXA regimens when assessing hemoglobin reduction (3.4 g/dL in the single-dose IV group and 3.6 g/dL in the double-dose IV group), calculated blood loss (*P* = 0.90), or transfusion rates (14% in the single-dose IV group and 18% in the double-dose IV group), whereas according to Kang et al. [34] three doses (3 g) of postoperative IV-TXA reduced blood loss and reduced postoperative inflammation and fibrinolytic reactions more than a single dose (1 g) or two doses (2 g) in elderly patients after TKA without increasing the incidence of adverse events [34]. A similar observation was made in the Chen et al. [35]; a similar view was reported in the study by Chen et al.; a recent randomized controlled trial study showed that [33] the single-dose regimen was non-inferior compared to the two-dose regimen. Therefore, this meta-analysis will answer the question of whether two-dose versus single-dose intravenous TXA is more effective in terms of total blood loss, transfusion rates, postoperative hemoglobin levels and length of hospital stay, deep venous thrombosis (DVT), and pulmonary embolism (PE), without sacrificing safety. Although some of the available studies reported that a single dose of TXA did not achieve effective outcomes and that 2 doses of TXA were the minimum dose required for effective outcomes with TKA, as the doses administered in the above studies required intraoperative dosing based on patient weight, they were not administered at a fixed dose (1 g fixed dose for 1 dose). Therefore, this meta-analysis compares the efficacy and safety of intravenous application of a fixed dose of TXA per dose after TKA and THA.

To the best of our knowledge, this is the first meta-analysis to compare the effectiveness and safety of fixed single and double doses of intravenous TXA in patients undergoing total hip arthroplasty or total hip replacement.

## Materials and methods

This meta-analysis was conducted in accordance with the recommendations of the Cochrane Handbook for the Systematic Evaluation of Interventions http://www.cochranehandbook.org and in accordance with the Prisma (preferred reporting entries for systematic evaluation and meta-analysis) checklist [36].

### Search strategy

Computer searches were conducted on PubMed, Medline, Embase, Web of Science, and The Cochrane Library from January 2000 to March 2023. The search included double doses of 1 g of IV TXA per dose and single doses of 1 g of IV TXA per dose to reduce TKA and THA. The search terms used were limited to the PubMed database, and the search results are shown in Additional file 1: Appendix Table S1. Publications were not restricted to English language literature, but article type was restricted to RCTs, including randomized controlled trial RCTs and cohort studies. Meta-analyses were performed to extract data from published papers. Literature selection, assessment of eligibility criteria, data extraction, and analysis of protocols registered in Prospero (CRD42023405387) were performed for our study protocols.

### Eligibility and exclusion criteria

#### The selection criteria are as follows

1. The study population was patients undergoing TKA and THA for the first time.
2. Study comparing intraoperative and postoperative indicators related to intravenous TXA double-dose and single-dose control of blood loss.
3. Outcome indicators include at least one of total blood loss; transfusion rate; postoperative hemoglobin; length of hospital stay; incidence in deep venous thrombosis; pulmonary embolism.
4. All clinical trial studies published observational studies, including RCTs, cohort studies.

#### The exclusion criteria are as follows

1. Literature with incomplete or no available data;
2. Non-intravenous route of administration and dose mismatch between the experimental and control groups;
3. Tests on cadavers or artificial models. Letters, reviews, editorials, systematic evaluations, and practice guidelines are also excluded.

### Data extraction and quality assessment

#### Data extraction

Two independent evaluators (Yong-Ze Yang and An-Ren Zhang) extracted and recorded the following data in a spreadsheet: patient demographics, author's name, date of publication, sample size, study site, body mass index, dose and timing of TXA application, and whether THA and TKA were initial unilateral or bilateral. Additionally, total blood loss, transfusion rate, postoperative hemoglobin, length of hospital stay, venous incidence of thromboembolism, and pulmonary artery embolism were recorded. All data were entered into a pre-generated Microsoft® Excel (Microsoft Corporation, Redmond, Washington, USA) spreadsheet. Data in other formats were converted according to Cochrane Handbook 5.0 guidelines. The data were then converted to mean ± standard deviation (SD) using the rules outlined in Cochrane Handbook 5.0.1 (http://www.cochrane-handbook.org/). In cases where information was incomplete, every effort was made to contact the author of the report to obtain it. If the information was ultimately unavailable, the literature was excluded.

#### Quality assessment of the included studies

Risk of bias was assessed according to the Cochrane Collaboration and the following criteria: random sequence generation, allocation concealment, participant blinding, outcome assessment blinding, incomplete outcome data, selective reporting, and other biases. Each item assessed as ‘yes’, ‘no’ or ‘unclear’ indicated low risk of bias, high risk of bias and lack of information or unknown risk of bias, respectively. For RCTs, the quality of the included randomized controlled studies (RCTs) was evaluated using the modified Jadad et al. scoring criteria in four main areas: (1) generation of randomized sequences: yes (2 points), unclear (1 point), no (0 points). (2) Whether the random grouping was hidden: yes (2 marks), unclear (1 mark), no (0 marks). (3) Whether blinding was used: yes (2 marks), unclear (1 mark), no (0 marks). (4) Withdrawal and withdrawal of cases or not: specific number and reason described (1 mark), no specific number or reason described (0 marks). Maximum 7 marks, where 1 to 3 marks are considered low quality and 4–7 marks are considered high quality.

The Newcastle–Ottawa Scale (NOS) was used to assess the quality of the included observational literature. The NOS assessment consisted of 3 items (9 points) specifically including selection of study subjects, comparability and outcome evaluation to assess selection, 4 items (4 points) to assess selection of study subjects for inclusion in the study, 1 item (2 points) to assess group comparability and 3 items (3 points) to assess outcomes of interest. The maximum score was 9. Studies scoring ≥ 6 were considered high quality, and those scoring < 6 were considered low quality. Two researchers independently completed the quality assessment of the included studies. Where inconsistencies existed, these were resolved by consulting the corresponding author.

### Statistical analysis

The main outcome indicators studied were: total blood loss, transfusion rate, postoperative hemoglobin, length of stay, incidence of deep venous thrombosis, and incidence of pulmonary embolism. Outcomes are expressed as mean differences (MD s) and 95% confidence intervals (CIs) for continuous outcomes (e.g., total blood loss and days in hospital). Dichotomous outcomes, such as incidence of transfusion rate, DVT and PE, were expressed as risk difference (RD) values with 95% CI statistical significance set at *P* < 0.05. To summarize the results of the trial, meta-analysis was performed using the software Rev Man 5.3 (Cochrane Collaboration Network, Oxford, UK). The Chi-square test and *I*^2^ statistics were used. Chi-square test results for *P* > 0.05 were considered to suggest statistical heterogeneity. If *I*^2^ < 50%, a fixed effects model was used; if *I*^2^ ≥ 50% was considered to indicate significant heterogeneity, a random effects model was used [37]. Publication bias and meta-regression were not assessable in the current meta-analysis, as tests for funnel plot asymmetry and meta-regression were usually only performed when at least 10 studies were included in the meta-analysis. There were only 5 studies in our meta-analysis; therefore, tests for asymmetry and meta-regression were not performed.

## Results

### Literature search results

The initial review yielded 1238 papers [(Pubmed (*n* = 239), Embase (*n* = 261), Web of Science (*n* = 385), Cochrane Library (*n* = 22), and Medline (*n* = 331)] after removing duplicates, and a total of 580 papers were screened. A total of 510 studies were excluded at the title and abstract level, and 5 studies were finally included by reading the full text and nadir criteria [28, 30, 38–40]. A total of 5542 patients were included for data extraction and meta-analysis. The five included studies were all cohort studies trials and all included studies were rated as high quality, all published after 2010, of which four included unilateral TKAs [20, 22, 31, 32], two included unilateral THRs [20, 32], and one studied bilateral TKA [30] with a total of 5542 patients, 3080 in the one dose 1 g TXA group and 2379 in the 2 dose 2 g TXA group; in both groups the mean age of the patients ranged from 62 to 75 years; transfusion criteria for inclusion in the study included less than 70 g/L, 80 g/L; and all patients included in the study received chemodynamic DVT prophylaxis such as rivaroxaban, low molecular weight heparin, or low molecular weight heparin and rivaroxaban.

**Fig. 1:**
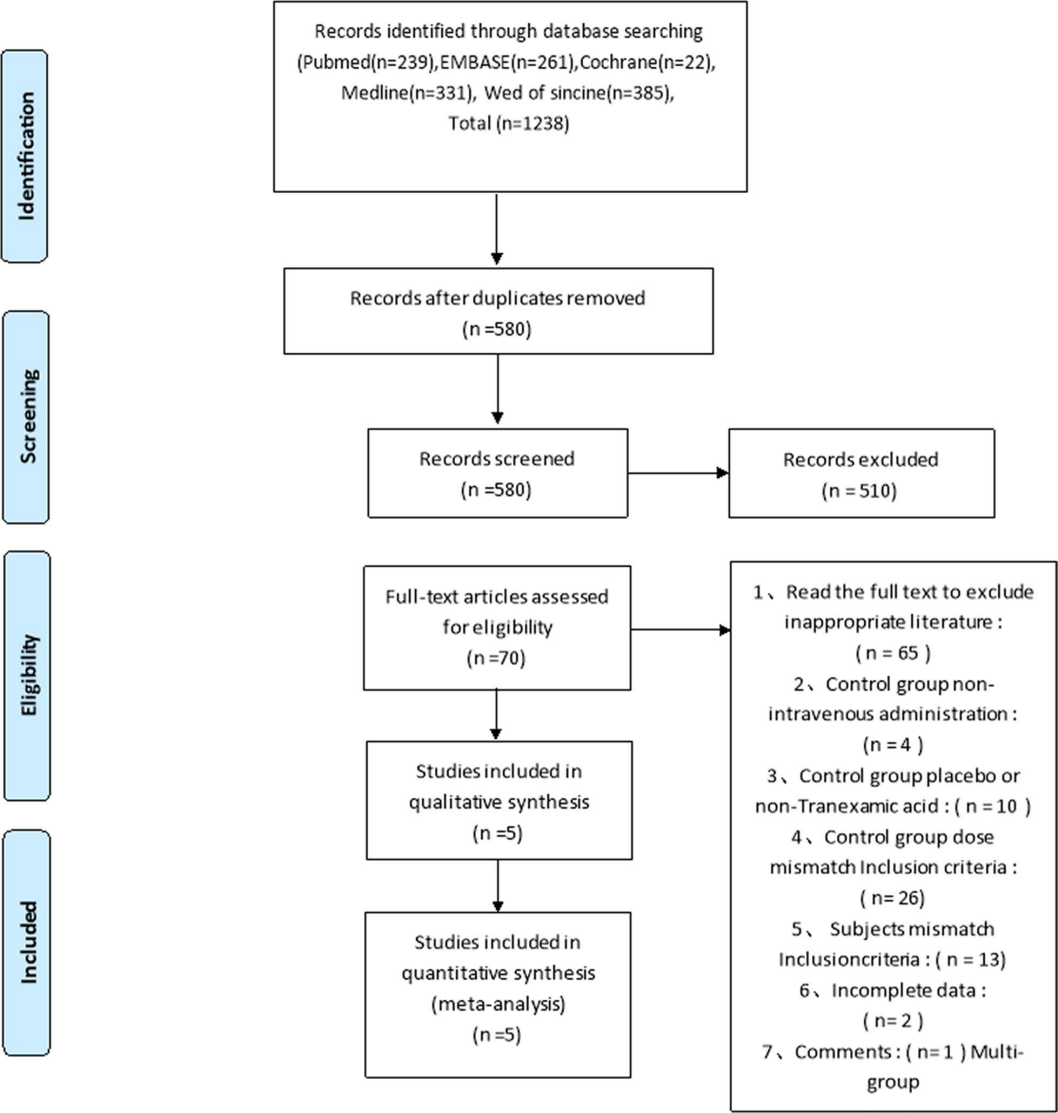
Flow chart of study inclusion and exclusion

The flow chart of study inclusion and exclusion is shown in Fig. 1.

The basic characteristics of the included studies are shown in Table 1.

**Table 1:** Basic characteristics of the entry study

Inclusion in the study	Country	Study design	Type of surgery	Number of participants	Age (mean ± SD)	BMI (kg/m^2^)	Blood transfusion standards (g/dl)	Dosing time		Number of cases (T/C)	Results
								T:1 g tranexamic acid group	C:2 g tranexamic acid group		
Takao/2013	Japan	Retrospective case–control study	Primary total knee arthroplasty	TKA: 47	S: 74.7 ± 5.3 D: 75.0 ± 5.0	S: 26.2 ± 4.2 D: 26.0 ± 3.1	≤ 8 g/dl	S: TXA IV (1 g); 10 min before bleeding of tourniquet	D: TXA IV (1 g) 10 min before and 3 h after tourniquet deflation	TKA: 21/26	1, 3, 5, 6
Wilde/2018	United States	Retrospective case–control study	Primary total knee arthroplasty and primary total hip arthroplasty	TKA: 1298 THR: 1144	TKA S: 69.5 ± 8.9 D: 68.9 ± 9.6 THR S: 67.4 ± 11.0 D: 66.9 ± 10.6	TKA S: 30.0 ± 6.5 D: 29.5 ± 6.0 THR S:30.0 ± 6.5 D: 29.5 ± 6.0	< 8.0 g/dL	S: 1 g push before cutting skin or 1 g push after tourniquet release	D: TXA by 1 g push before skin incision and 1 g push 6 h after the first dose or 1 g push after tourniquet release and 1 g push 6 h after the first dose (TKA)	TKA: 499/799 THR: 454/690	2, 3, 4, 5, 6
Charette /2019	United States	Retrospective case–control study	Primary total knee arthroplasty	TKA:1191	S: 62.6 D: 62	S: 34.3 D: 35	Not provided	S: IV 1 g TXA before incision	D: IV 1 g TXA before incision, another group IV1 gram TXA at wound closure	TKA: 891/354	1, 2, 4, 5, 6
Golz/2021	United States	Retrospective case–control study	Primary total knee arthroplasty and primary total hip arthroplasty	TKA: 928 THR: 592	S: 65.4 (10.2) D: 64.2 (9.83)	S: 32.6 ± (6.8) D: 32.7 ± (7.0)	≤ 7.0 g/dL	S: IV 1 g TXA before incision	Group D: IV 1 g TXA before incision, another group IV 1 g TXA at wound closure	TKA: 407/521 THR: 466/126	2, 3, 4, 5, 6
Wilde/2022	United States	Retrospective case–control study	Primary simultaneous bilateral total knee arthroplasty	TKA: 259	S: 65 ± 7.8 D: 67 ± 7	S: 30 ± 6 D: 31 ± 1	< 8.0 g/dL	S: 1 g of IV TXA immediately after tourniquet release	D: IV 1 g TXA immediately after tourniquet release and IV 1 g TXA 6 h after initial dosing	TKA:98/161	2, 3, 5, 6,

(1) Total blood loss (TBL); (2) transfusion rate; (3) postoperative hemoglobin; (4) length of hospital stay; (5) incidence of symptomatic venous thromboembolism; (6) pulmonary artery embolism

### Evaluation of the methodological quality of the included studies

The results of the cohort study of 5 articles are presented in Table 2 at jurisprudence quality evaluation.

**Table 2:** Quality evaluation results of non-randomized controlled studies

Inclusion in the study	Selection of research subjects				Comparability	Outcome measurement			Rating
	1	2	3	4		A	B	C	
Takao/2013	*	*	*	*	*	*		*	7
Wilde/2018	*	*	*	*	*	*		*	7
Charette/2019	*	*	*	*	*	*		*	7
Golz/2021	*	*	*	*	*	*		*	7
Wilde/2022	*	*	*	*	*	*		*	7

(1) Representativeness of the exposure cohort; (2) Selection of unexposed; (3) Determination of exposure; (4) Outcomes not present at the start; (A) Outcome assessment; (B) Adequate follow-up time; (C) Adequacy of follow-up

### Meta-analysis results

#### Post-operative blood loss

**Fig. 2:**
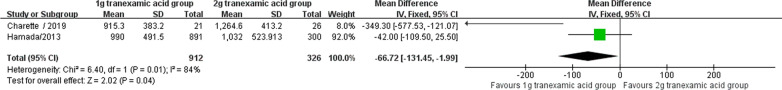
Postoperative blood loss

Two studies [22, 30] (1228 patients) reported data on total blood loss. Therefore, we included them as data for meta-analysis (*P* = 0.01; *I*^2^ = 83%). There was significant between-study heterogeneity; therefore, a random-effects model was used for analysis. The results showed no difference between 1 and 2 doses in total blood loss (MD = − 65.60, 95% CI [− 131.46, 0.26], *P* = 0.05, Fig. 2).

#### Blood transfusion rate

**Fig. 3:**
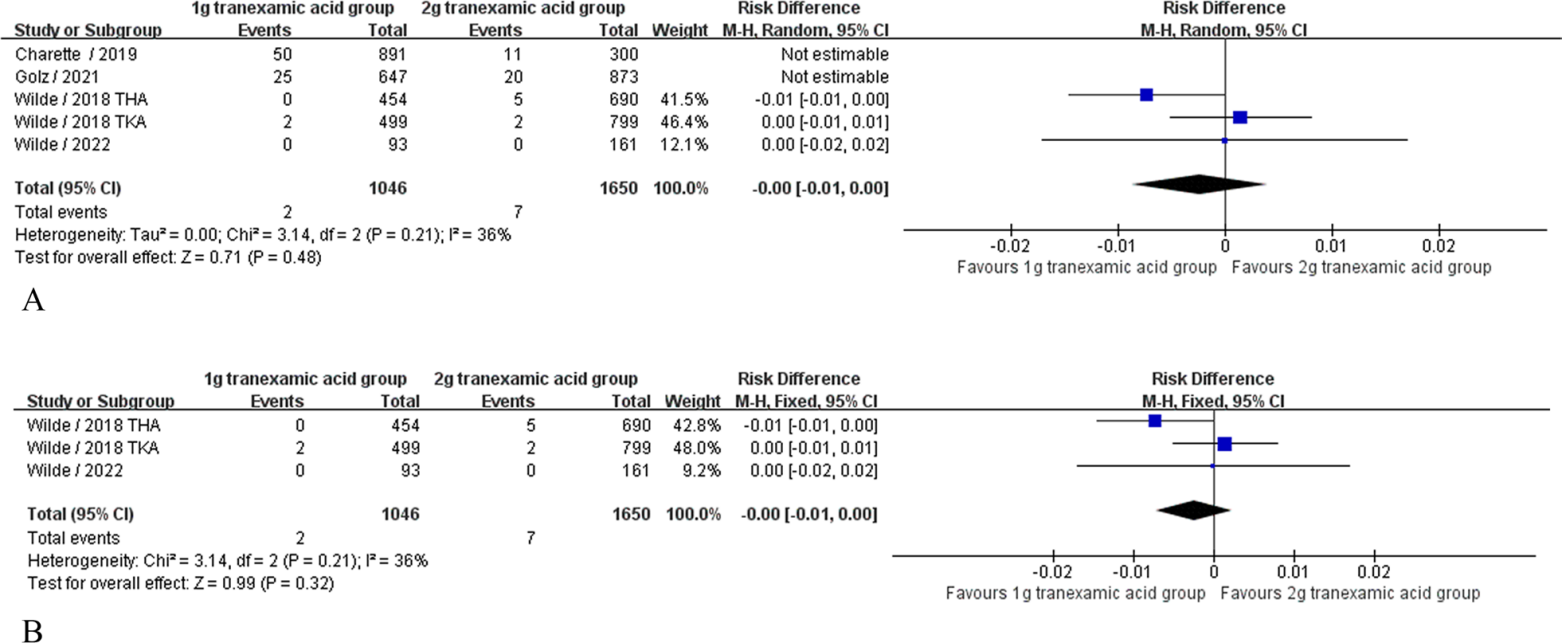
**A** Blood transfusion rate. **B** Blood transfusion rate sensitivity analysis

Four articles [20, 30–32] (5407 patients) reported outcomes for postoperative transfusion rates. Heterogeneity between studies was significant (*P* = 0.0005; *I*^2^ = 80%); therefore, a random effects model was used for analysis. Pooled results showed no significant difference in postoperative transfusion rates between the 1-dose, 1 g TXA group and the 2-dose, 2 g TXA group (RD = 0.00, 95% CI [− 0.01, 0.02], *P* = 0.55, Fig. 3A). Sensitivity analysis was performed and found that after excluding the Charette 2019 and Golz 2021gramroups of trials [39, 40], although the point estimates were in the same direction; the results of the remaining three groups still showed no difference between the two groups, but heterogeneity *I*^2^ from 80 to 36% (RD = 0.00, 95% CI [− 0.01, 0.02], *P* = 0.32, Fig. 3B).

#### Postoperative hemoglobin

**Fig. 4:**
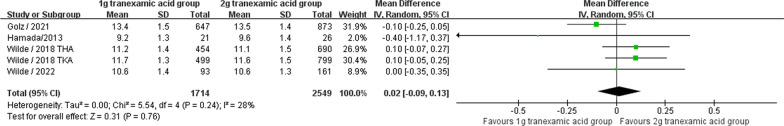
Post-operative hemoglobin

Four articles [20, 22, 30, 32] (4263 patients) reported outcomes of postoperative hemoglobin decline. Inter-study heterogeneity was low (*P* = 0.24; *I*^2^ = 28%); therefore, a fixed-effects model was performed for analysis. Pooled results showed no significant difference in postoperative hemoglobin decline between the 1-dose, 1 g TXA group and the 2-dose, 2 g TXA group (MD = 0.02, 95% CI [− 0.09, 0.13], *P* = 0.31, Fig. 4).

#### Length of stay in hospital

**Fig. 5:**
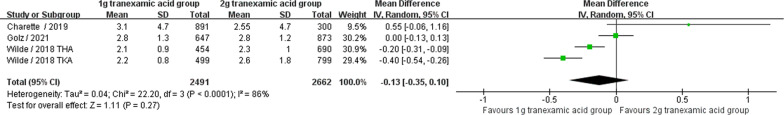
Length of stay in hospital

Three articles [20, 31, 32] (5243 patients) reported patient outcomes for postoperative length of stay. Heterogeneity between studies was significant (*P* = 0.0001; *I*^2^ = 86%); therefore, a random effects model was performed for analysis. Pooled results showed no significant difference in the number of days of postoperative hospital stay between the 1-dose 1 g TXA group and the 2-dose 2 g TXA group (MD = − 0.13), 95% CI [− 0.35, 0.09], *P* = 0.25, Fig. 5). Sensitivity analysis was performed and found that the point estimates were in the same direction, indicating that the study as a whole was relatively stable.

#### Deep venous thrombosis

**Fig. 6:**
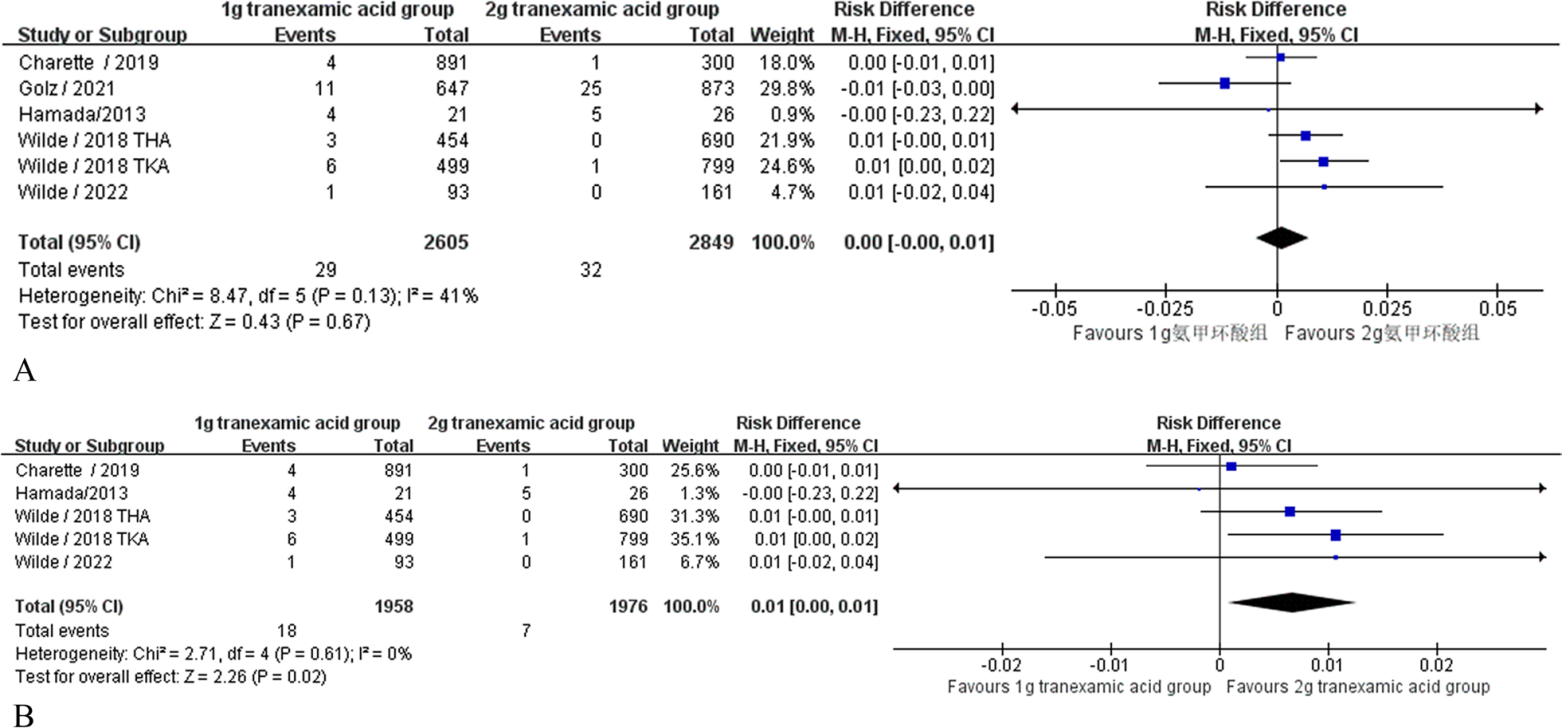
**A** Deep venous thrombosis. **A** Deep venous thrombosis sensitivity analysis

Five articles [28, 30, 38–40] (5454 patients) reported the incidence of deep vein thrombosis. No significant heterogeneity was found (*P* = 0.13; *I*^2^ = 41%), so a fixed-effects model was used. The difference between the two groups was not statistically significant (RD = 0.00, 95% CI [− 0.00, 0.01], *P* = 0.67, Fig. 6A). When sensitivity analyses were performed, after excluding one trial Golz 2021 the point estimates and 95% CI given in the sensitivity analysis of the fixed effects model were found to favor the 1 g group tranexamic acid in reducing the incidence of deep vein thrombosis after knee replacement (RD = 0.01, 95% CI [0.00, 0.01], *P* = 0.02 and *1*^2^ = 0% Fig. 6B) [40].

#### Pulmonary embolism

**Fig. 7:**
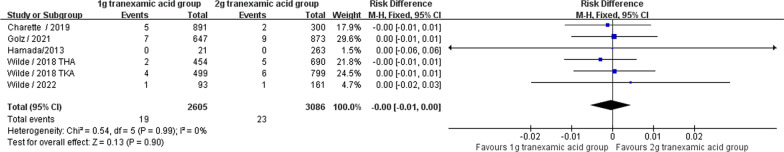
Pulmonary embolism

Five of the included [28, 30, 38–40] studies (5691 patients) reported no significant heterogeneity in pulmonary embolism (*P* = 0.97; *I*^2^ = 0%); therefore, a fixed-effects model was used. There was no significant difference between them (RD = 0.00, 95% CI [− 0.01, 0.00], *P* = 0.79; Fig. 7).

## Discussion

TXA has been used in TKA and THA for a long time with positive clinical results. When TXA is applied intravenously, it is widely distributed extracellularly and intracellularly and diffuses into the joint fluid and synovial membranes, reaching the same concentration as serum in the joint fluid [11, 20, 41]. The biological half-life of TXA in the joint fluid is approximately 3 h. In addition, studies have shown that [19] that fibrinolysis peaks 6 h after the end of surgery and is maintained for approximately 18 h after hip and knee arthroplasty. The antifibrinolytic properties of this substance have resulted in its widespread use in major arthroplasties. However, due to the limited number of published studies on double and single fixed doses in primary hip and knee arthroplasty, we excluded published randomized controlled trials on single and double fixed doses during the screening process, as they were conducted on patients undergoing revision arthroplasty. Although we ultimately included only cohort studies, the large sample size of the included studies made our meta-analysis more credible. Based on the available evidence, the most important finding of our meta-analysis was that there was no significant difference between the two dosing regimens of TXA. Single- and double-dose intravenous administration of TXA had similar effects in terms of reducing blood loss, transfusion rates, postoperative hemoglobin levels, and length of hospital stay, without increasing the risk of complications such as DVT or PE.

A large body of literature suggests that the use of TXA is associated with satisfactory outcomes in patients undergoing TKA and THA [42]. However, there is no uniform consensus on the dose of TXA used intravenously in THA and TKA in the studies that have been published. Despite considerable progress in perioperative management, blood loss and transfusion remain an issue of concern in arthroplasty [43]. Patients undergoing TKA and THA are at risk of perioperative blood loss. Many require allogeneic blood transfusions without the use of antifibrinolytic drugs, which can lead to adverse effects associated with anemia and allogeneic blood [44] and may even be life-threatening, such as immune reactions, infection, hemolysis, and additional financial burden. In this meta-analysis, all but one study did not mention transfusion rates [30]. In this meta-analysis, all but one study did not mention transfusion rates, while the other four studies mentioned transfusion at [20, 30–32].

The results of the meta-analysis indicate that there was no significant difference in the blood transfusion rate between the double-dose and single-dose groups of tranexamic acid, suggesting that different doses of tranexamic acid have no effect on the postoperative blood transfusion rate of patients. However, upon careful examination of the included studies, it was found that the blood transfusion criteria varied among the studies, which may have affected the final results. Additionally, the number of studies included was small, and further studies are needed to provide a more comprehensive explanation. It is important to note that differences in blood transfusion standards can also affect the blood transfusion rate. Charette et al. [27] reported that high blood transfusion rates may be due to the lack of reference to blood transfusion standards in cohort studies. Furthermore, although the anesthesia method was not included as an outcome indicator for evaluation, it has been reported that the anesthesia method can affect blood loss and blood transfusion requirements. According to Jonathan et al. [33], general anesthesia is positively correlated with the occurrence of adverse events and blood transfusion rate compared to spinal anesthesia. However, this conclusion has not been widely recognized, and large sample size and multicenter studies are needed to confirm the conclusion.

The duration of hospitalization is a significant predictor of the overall cost of total joint replacement surgery. The interest in hospitalization duration has led to the development of the concept of recovery after surgery (ERAS) [45, 46]. Some of the literature reviewed in this study suggests that a double dose of TXA can reduce the duration of hospitalization after hip and knee arthroplasty. However, the combined results showed no significant difference in hospitalization duration between double and single doses. Additionally, Charette et al. [39] found that the two-dose TXA regimen significantly increased the risk of readmission (OR = 3.14; *P* < 0.001) and reoperation (OR = 3.65; *P* = 0.034) compared to the single-dose regimen in unilateral primary TKA. The two-dose TXA regimen also reduced the duration of hospital stay. However, only three studies in the meta-analysis reported hospitalization duration [28, 39, 40], so the combined results may be controversial. Further studies are needed to confirm these findings.

Due to the mechanism of action of tranexamic acid (TXA) in total knee arthroplasty (TKA) and total hip arthroplasty (THA), there is a high risk of thrombotic complications when utilizing its antifibrinolytic effects, which can lead to serious complications such as deep vein thrombosis (DVT) and pulmonary embolism (PE). Therefore, it is important to determine whether multiple intravenous applications of TXA increase the incidence of thrombotic events. In this meta-analysis, no significant differences in DVT and PE were found between double-dose intravenous TXA and single-dose intravenous TXA after hip and knee arthroplasty. All included studies administered early postoperative chemoprophylaxis to prevent thrombotic complications, which may account for the lower incidence of DVT and PE. However, sensitivity analyses for DVT found that after excluding one trial [40], the fixed effects model favored the use of 1 g of TXA to reduce the incidence of DVT after knee replacement. The source of heterogeneity may be related to the fact that this study used different anticoagulation measures in patients postoperatively. Additionally, the short follow-up period of the included studies may have underestimated the incidence of thrombotic complications. Therefore, larger, well-designed randomized controlled trials with longer follow-up periods are needed to assess the safety and efficacy of double-dose intravenous TXA application versus single-dose intravenous TXA application in regards to the incidence of DVT and PE.

The body's natural antifibrinolytic processes after joint replacement surgery can reduce the efficacy of the second dose [41, 47]. In accordance with this theory, our study showed no difference in postoperative hemoglobin levels between the one- and two-dose regimens. Our results are in line with Wilde et al. [28] in agreement with Wilde et al. demonstrating that single-dose TXA is as effective as two-dose TXA in reducing blood loss and transfusion rates after THA and TKA without difference and without altering the incidence of deep venous thrombosis and pulmonary embolism complications. And the use of single-dose therapy reduces costs. If the Moskal et al. [48] estimated a price of $39.14 for a single 1 g IV TXA dose and $78.28 for two doses for 1,563,421 doses implemented in 2020, mainly TKAs and THAs and if all patients received a single dose instead of two, this would save approximately $61.2 million this year. In our study, which was scored according to NOS criteria, most of the literature was of high quality, which makes the conclusions more reliable, but we should not ignore various limitations presented in our MATE.

### Limitations

There are some limitations to this meta-analysis. (1) Some of the included studies transfusion index methods were not described, which may affect the results; (2) The timing of TXA administration was different between the two groups of studies with single and double doses, which may affect the MATE results; (3) The meta-analysis included four TKA studies, two THA studies, and one bilateral TKA study. The differences in the two operation methods may affect the results; (4) The differences in operation time, technique, access, and prosthesis type of the included studies may have an impact on the results; (5) Different anesthesia methods may affect the amount of intraoperative bleeding and blood transfusion; (6) Postoperative prevention of DVT and PE varies between the included studies; (7) Short-term follow-up may underestimate the incidence of complications, including DVT and PE; therefore, future studies should also focus on other factors of TXA in TKA and THA that may affect postoperative outcomes. This study aimed to TXA in the study of knee arthroplasty and hip arthroplasty, but only two studies in the literature reporting on the hip met our inclusion criteria, so more studies reporting on how TXA is used in the hip are needed to further illustrate the effectiveness and safety of TXA in arthroplasty.

## Conclusion

The use of single-dose and double-dose TXA in THA and TKA surgery has comparable effects in reducing blood loss, transfusion rate, postoperative hemoglobin levels, and length of hospital stay, without increasing the risk of complications such as DVT or PE. Therefore, single-dose TXA is recommended. However, larger, well-designed, and high-quality randomized controlled trials with long-term follow-up are still necessary to confirm these findings.

### Supplementary Information

**Additional file 1.** **Annex 1:** All search formulas of literature.

## Data Availability

Data sharing is not applicable.

## Abbreviations

TXA: Tranexamic acid
MD: Mean difference
IV: Intravenous
THA: Total hip arthroplasty
TKA: Total knee arthroplasty
DVT: Deep venous thrombosis
PE: Pulmonary embolism
CI: Confidence interval
RD: Risk difference
