# Supplementary material for: Efficacy and safety of single- and double-dose intravenous tranexamic acid in hip and knee arthroplasty: a systematic review and meta-analysis
Source: J Orthop Surg Res. 2023 Aug 10;18:593. doi: 10.1186/s13018-023-03929-9 (PMC10413625; doi:10.1186/s13018-023-03929-9)
Supplement: Supplementary file 1 — Additional file 1. Annex 1: All search formulas of literature. [file 13018_2023_3929_MOESM1_ESM.docx]

**PUBMED**

| **Search** | **Query** | **Results** |
| --- | --- | --- |
| # 1 | (Arthroplasties, Replacement, Knee[Title/Abstract] OR Arthroplasty, Knee Replacement[Title/Abstract] OR Knee Replacement Arthroplasties[Title/Abstract] OR Knee Replacement Arthroplasty[Title/Abstract] OR Replacement Arthroplasties, Knee[Title/Abstract] OR Knee Arthroplasty, Total[Title/Abstract] OR Arthroplasty, Total Knee[Title/Abstract] OR Total Knee Arthroplasty[Title/Abstract] OR Replacement, Total Knee[Title/Abstract] OR Total Knee Replacement[Title/Abstract] OR Knee Replacement, Total[Title/Abstract] OR Knee Arthroplasty[Title/Abstract] OR Arthroplasty, Knee[Title/Abstract] OR Arthroplasties, Knee Replacement[Title/Abstract] OR Replacement Arthroplasty, Knee[Title/Abstract] OR knee joint replacement[Title/Abstract] OR knee joint replacements [Title/Abstract]OR "Arthroplasty, Replacement, Knee"[Mesh]) | 47,652 |
| # 2 | (Arthroplasties, Replacement, Hip[Title/Abstract] OR Arthroplasty, Hip Replacement[Title/Abstract] OR Hip Replacement Arthroplasties[Title/Abstract] OR Hip Prosthesis Implantation[Title/Abstract] OR Hip Prosthesis Implantations[Title/Abstract] OR Implantation, Hip Prosthesis[Title/Abstract] OR Prosthesis Implantation, Hip[Title/Abstract] OR Replacement Arthroplasties, Hip[Title/Abstract] OR Replacement Arthroplasty, Hip[Title/Abstract] OR Arthroplasties, Hip Replacement[Title/Abstract] OR Hip Replacement Arthroplasty[Title/Abstract] OR Hip Replacement, Total[Title/Abstract] OR Replacement, Total Hip[Title/Abstract] OR Total Hip Replacements[Title/Abstract] OR Total Hip Replacement[Title/Abstract] OR Total Hip Arthroplasty[Title/Abstract] OR Arthroplasty, Total Hip[Title/Abstract] OR Hip Arthroplasty, Total[Title/Abstract] OR Total Hip Arthroplasties[Title/Abstract] OR "Arthroplasty, Replacement, Hip"[Mesh]) | 47,313 |
| # 3 | #1 OR #2 | 85,989 |
| # 4 | (Doses[Title/Abstract]) OR (Dose[Title/Abstract]) | 1,480,030 |
| #5 | ((((((((((((AMCHA[Title/Abstract]) OR (trans-4-(Aminomethyl)cyclohexanecarboxylic Acid[Title/Abstract])) OR (t-AMCHA[Title/Abstract])) OR (AMCA[Title/Abstract])) OR (Anvitoff[Title/Abstract])) OR (Cyklokapron[Title/Abstract])) OR (Ugurol[Title/Abstract])) OR (KABI 2161[Title/Abstract])) OR (Spotof[Title/Abstract])) OR (Transamin[Title/Abstract])) OR (Amchafibrin[Title/Abstract])) OR (Exacyl[Title/Abstract])) OR ("Tranexamic Acid"[Mesh]) | 5186 |
| #6 | #3 AND #4 AND #5 | 239 |

WEB OF SICENE

| **Search** | **Query** | **Results** |
| --- | --- | --- |
| # 1 | ((((((((((((((((((TS=(Arthroplasty, Replacement, Knee)) OR TS=(Arthroplasties, Replacement, Knee)) OR TS=(Arthroplasty, Knee Replacement)) OR TS=(Knee Replacement Arthroplasties)) OR TS=(Knee Replacement Arthroplasty)) OR TS=(Replacement Arthroplasties, Knee)) OR TS=(Knee Arthroplasty, Total)) OR TS=(Arthroplasty, Total Knee)) OR TS=(Total Knee Arthroplasty)) OR TS=(Replacement, Total Knee)) OR TS=(Total Knee Replacement)) OR TS=(Knee Replacement, Total)) OR TS=(Knee Arthroplasty)) OR TS=(Arthroplasty, Knee)) OR TS=(Arthroplasties, Knee Replacement)) OR TS=(Replacement Arthroplasty, Knee)) OR TS=(knee joint replacement)) OR TS=(knee joint replacements)) OR TS=(Replacement Arthroplasty, Knee) | 56,032 |
| # 2 | (((((((((((((((((((TS=(Arthroplasties, Replacement, Hip)) OR TS=(Arthroplasty, Hip Replacement)) OR TS=(Hip Replacement Arthroplasties)) OR TS=(Hip Prosthesis Implantation)) OR TS=(Hip Prosthesis Implantations)) OR TS=( Implantation, Hip Prosthesis)) OR TS=(Prosthesis Implantation, Hip)) OR TS=(Replacement Arthroplasties, Hip)) OR TS=(Replacement Arthroplasty, Hip)) OR TS=(Arthroplasties, Hip Replacement)) OR TS=( Hip Replacement Arthroplasty)) OR TS=(Hip Replacement, Total)) OR TS=(Replacement, Total Hip)) OR TS=(Total Hip Replacements)) OR TS=(Total Hip Replacement)) OR TS=(Total Hip Arthroplasty)) OR TS=(Arthroplasty, Total Hip)) OR TS=(Hip Arthroplasty, Total)) OR TS=(Total Hip Arthroplasties)) OR TS=(Arthroplasty, Replacement, Hip) | 60,042 |
| # 3 | (TS=(Doses)) OR TS=(Dose) | 501,335 |
| # 4 | ((((((((((((TS=(AMCHA)) OR TS=(trans-4-(Aminomethyl)cyclohexanecarboxylic Acid)) OR TS=(t-AMCHA)) OR TS=(AMCA)) OR TS=(Anvitoff)) OR TS=(Cyklokapron)) OR TS=(Ugurol)) OR TS=(KABI 2161)) OR TS=(Spotof)) OR TS=(Transamin)) OR TS=(Amchafibrin)) OR TS=(Exacyl)) OR TS=(Tranexamic Acid) | 8734 |
| #5 | #1 OR #2 | 92509 |
| #6 | #3 AND #4 AND #5 | 385 |

**MEDLINE**

| **Search** | **Query** | **Results** |
| --- | --- | --- |
| # 1 | ((((((((((((((((((TS=(Arthroplasty, Replacement, Knee)) OR TS=(Arthroplasties, Replacement, Knee)) OR TS=(Arthroplasty, Knee Replacement)) OR TS=(Knee Replacement Arthroplasties)) OR TS=(Knee Replacement Arthroplasty)) OR TS=(Replacement Arthroplasties, Knee)) OR TS=(Knee Arthroplasty, Total)) OR TS=(Arthroplasty, Total Knee)) OR TS=(Total Knee Arthroplasty)) OR TS=(Replacement, Total Knee)) OR TS=(Total Knee Replacement)) OR TS=(Knee Replacement, Total)) OR TS=(Knee Arthroplasty)) OR TS=(Arthroplasty, Knee)) OR TS=(Arthroplasties, Knee Replacement)) OR TS=(Replacement Arthroplasty, Knee)) OR TS=(knee joint replacement)) OR TS=(knee joint replacements)) OR TS=(Replacement Arthroplasty, Knee) | 50,309 |
| # 2 | (((((((((((((((((((TS=(Arthroplasties, Replacement, Hip)) OR TS=(Arthroplasty, Hip Replacement)) OR TS=(Hip Replacement Arthroplasties)) OR TS=(Hip Prosthesis Implantation)) OR TS=(Hip Prosthesis Implantations)) OR TS=( Implantation, Hip Prosthesis)) OR TS=(Prosthesis Implantation, Hip)) OR TS=(Replacement Arthroplasties, Hip)) OR TS=(Replacement Arthroplasty, Hip)) OR TS=(Arthroplasties, Hip Replacement)) OR TS=( Hip Replacement Arthroplasty)) OR TS=(Hip Replacement, Total)) OR TS=(Replacement, Total Hip)) OR TS=(Total Hip Replacements)) OR TS=(Total Hip Replacement)) OR TS=(Total Hip Arthroplasty)) OR TS=(Arthroplasty, Total Hip)) OR TS=(Hip Arthroplasty, Total)) OR TS=(Total Hip Arthroplasties)) OR TS=(Arthroplasty, Replacement, Hip) | 54,290 |
| # 3 | (TS=(Doses)) OR TS=(Dose) | 1,814,673 |
| #4 | ((((((((((((TS=(AMCHA)) OR TS=(trans-4-(Aminomethyl)cyclohexanecarboxylic Acid)) OR TS=(t-AMCHA)) OR TS=(AMCA)) OR TS=(Anvitoff)) OR TS=(Cyklokapron)) OR TS=(Ugurol)) OR TS=(KABI 2161)) OR TS=(Spotof)) OR TS=(Transamin)) OR TS=(Amchafibrin)) OR TS=(Exacyl)) OR TS=(Tranexamic Acid) | 7402 |
| #5 | #1 OR #2 | 91,270 |
| #6 | #3 AND #4 AND #5 | 331 |

COCHRAN

| **Search** | **Query** | **Results** |
| --- | --- | --- |
| # 1 | MeSH descriptor: [Arthroplasty, Replacement, Knee] explode all trees | 3343 |
| # 2 | (Arthroplasty, Replacement, Knee):ti,ab,kw OR (Arthroplasties, Replacement, Knee):ti,ab,kw OR (Arthroplasty, Knee Replacement):ti,ab,kw OR (Knee Replacement Arthroplasties):ti,ab,kw OR (Knee Replacement Arthroplasty):ti,ab,kw OR (Replacement Arthroplasties, Knee):ti,ab,kw OR (Knee Arthroplasty, Total):ti,ab,kw OR (Arthroplasty, Total Knee):ti,ab,kw OR (Total Knee Arthroplasty):ti,ab,kw OR (Replacement, Total Knee):ti,ab,kw OR (Total Knee Replacement):ti,ab,kw OR (Knee Replacement, Total):ti,ab,kw OR (Knee Arthroplasty):ti,ab,kw OR (Arthroplasty, Knee):ti,ab,kw OR (Arthroplasties, Knee Replacement):ti,ab,kw OR (Replacement Arthroplasty, Knee):ti,ab,kw OR (knee joint replacement):ti,ab,kw OR (knee joint replacements):ti,ab,kw | 9932 |
| # 3 | #1 OR #2 | 9932 |
| # 4 | MeSH descriptor: [Arthroplasty, Replacement, Hip] explode all trees | 2363 |
| #5 | (Arthroplasties, Replacement, Hip):ti,ab,kw OR (Arthroplasty, Hip Replacement):ti,ab,kw OR (Hip Replacement Arthroplasties):ti,ab,kw OR (Hip Prosthesis Implantation):ti,ab,kw OR (Hip Prosthesis Implantations):ti,ab,kw OR ( Implantation, Hip Prosthesis):ti,ab,kw OR (Prosthesis Implantation, Hip):ti,ab,kw OR (Replacement Arthroplasties, Hip):ti,ab,kw OR (Replacement Arthroplasty, Hip):ti,ab,kw OR (Arthroplasties, Hip Replacement):ti,ab,kw OR ( Hip Replacement Arthroplasty):ti,ab,kw OR (Hip Replacement, Total):ti,ab,kw OR (Replacement, Total Hip):ti,ab,kw OR (Total Hip Replacements):ti,ab,kw OR (Total Hip Replacement):ti,ab,kw OR (Total Hip Arthroplasty):ti,ab,kw OR (Arthroplasty, Total Hip):ti,ab,kw OR (Hip Arthroplasty, Total):ti,ab,kw OR (Total Hip Arthroplasties):ti,ab,kw | 7235 |
| #6 | #4 OR #5 | 7235 |
|  | (Doses):ti,ab,kw OR (Dose):ti,ab,kw | 3364169 |
| #7 | MeSH descriptor: [Tranexamic Acid] explode all trees | 1497 |
| #8 | (AMCHA):ti,ab,kw OR (trans-4-(Aminomethyl)cyclohexanecarboxylic Acid):ti,ab,kw OR (t-AMCHA):ti,ab,kw OR (AMCA):ti,ab,kw OR (Anvitoff):ti,ab,kw OR (Cyklokapron):ti,ab,kw OR (Ugurol):ti,ab,kw OR (KABI 2161):ti,ab,kw OR (Spotof):ti,ab,kw OR (Transamin):ti,ab,kw OR (Amchafibrin):ti,ab,kw OR (Exacyl):ti,ab,kw | 127 |
| #9 | #8 OR #9 | 1596 |
| #10 | #3 AND #6 AND #7 AND #10 | 22 |

**EMBASE**

| No. | Query | Results |
| --- | --- | --- |
| #59 | #41 AND #45 AND #58 | 261 |
| #58 | #46 OR #47 OR #48 OR #49 OR #50 OR #51 OR #52 OR #53 OR #54 OR #55 OR #56 OR #57 | 19036 |
| #57 | exacyl | 159 |
| #56 | amchafibrin | 40 |
| #55 | transamin | 122 |
| #54 | kabi AND 2161 | 7 |
| #53 | ugurol | 79 |
| #52 | cyklokapron | 313 |
| #51 | anvitoff | 38 |
| #50 | amca | 520 |
| #49 | 't amcha' | 52 |
| #48 | 'trans 4 aminomethyl cyclohexanecarboxylic' AND acid | 23 |
| #47 | amcha | 166 |
| #46 | tranexamic AND acid | 18510 |
| #45 | #43 OR #44 | 2869305 |
| #44 | dose | 2616423 |
| #43 | doses | 707554 |
| #42 | #20 OR #41 | 114326 |
| #41 | #21 OR #22 OR #23 OR #24 OR #25 OR #26 OR #27 OR #28 OR #29 OR #30 OR #31 OR #32 OR #33 OR #34 OR #35 OR #36 OR #37 OR #38 OR #39 OR #40 | 63309 |
| #40 | arthroplasty, AND replacement, AND hip | 25192 |
| #39 | total AND hip AND arthroplasties | 5499 |
| #38 | hip AND arthroplasty, AND total | 46541 |
| #37 | arthroplasty, AND total AND hip | 46541 |
| #36 | total AND hip AND arthroplasty | 46541 |
| #35 | total AND hip AND replacement | 32492 |
| #34 | total AND hip AND replacements | 4238 |
| #33 | replacement, AND total AND hip | 32492 |
| #32 | hip AND replacement, AND total | 32492 |
| #31 | hip AND replacement AND arthroplasty | 25192 |
| #30 | arthroplasties, AND hip AND replacement | 2175 |
| #29 | replacement AND arthroplasty, AND hip | 25192 |
| #28 | replacement AND arthroplasties, AND hip | 2175 |
| #27 | prosthesis AND implantation, AND hip | 4263 |
| #26 | implantation, AND hip AND prosthesis | 4263 |
| #25 | hip AND prosthesis AND implantations | 194 |
| #24 | hip AND prosthesis AND implantation | 4263 |
| #23 | hip AND replacement AND arthroplasties | 2175 |
| #22 | arthroplasty, AND hip AND replacement | 25192 |
| #21 | arthroplasties, AND replacement, AND hip | 2175 |
| #20 | #1 OR #2 OR #3 OR #4 OR #5 OR #6 OR #7 OR #8 OR #9 OR #10 OR #11 OR #12 OR #13 OR #14 OR #15 OR #16 OR #17 OR #18 OR #19 | 69854 |
| #19 | knee AND joint AND replacements | 2491 |
| #18 | knee AND joint AND replacement | 17649 |
| #17 | replacement AND arthroplasty, AND knee | 23956 |
| #16 | arthroplasties, AND knee AND replacement | 1565 |
| #15 | arthroplasty, AND knee | 63233 |
| #14 | knee AND arthroplasty | 63233 |
| #13 | knee AND replacement, AND total | 23622 |
| #12 | total AND knee AND replacement | 23622 |
| #11 | total AND knee AND arthroplasty | 50845 |
| #10 | replacement, AND total AND knee | 23622 |
| #9 | arthroplasty, AND total AND knee | 50845 |
| #8 | knee AND arthroplasty, AND total | 50845 |
| #7 | replacement AND arthroplasties, AND knee | 1565 |
| #6 | knee AND replacement AND arthroplasty | 23956 |
| #5 | knee AND replacement AND arthroplasties | 1565 |
| #4 | arthroplasty, AND knee AND replacement | 23956 |
| #3 | arthroplasties, AND replacement, AND knee | 1565 |
| #2 | arthroplasty, AND replacement, AND knee | 23956 |
| #1 | ('replacement'/exp OR replacement) AND ('arthroplasty,'/exp OR arthroplasty,) AND ('knee'/exp OR knee) | 26438 |
